# High expression of TAZ indicates a poor prognosis in retinoblastoma

**DOI:** 10.1186/s13000-015-0415-9

**Published:** 2015-10-13

**Authors:** Yiting Zhang, Chunyan Xue, Hongjuan Cui, Zhenping Huang

**Affiliations:** Department of Ophthalmology, Medical School of Nanjing University, Jinling Hospital, Nanjing, 210002 China; State Key Laboratory of Silkworm Genome Biology, Institute of Sericulture and Systems Biology, Southwest University, Chongqing, 400716 China

**Keywords:** TAZ, Retinoblastoma, Prognosis, Proliferation

## Abstract

**Background:**

The transcriptional co-activator, TAZ, is an important effector of the Hippo pathway and is critical for the development of human cancers. However, the expression and prognostic significance of TAZ in retinoblastoma is currently unclear.

**Methods:**

TAZ expression was examined in 43 retinoblastoma samples by immunohistochemistry. The relationship between TAZ expression and the clinicopathological features of retinoblastoma was also analyzed. Cox regression and Kaplan-Meier survival analyses were used to identify the prognostic factors for retinoblastoma patients. Finally, the effects of TAZ on cell proliferation were explored through lentivirus-mediated downregulation of TAZ in retinoblastoma cells.

**Results:**

TAZ was highly expressed in retinoblastoma tissues and was associated with regional lymph node classification (*P* = 0.013), largest tumor base (*P* = 0.045), and differentiation (*P* = 0.019). Moreover, patients with high TAZ expression had shorter overall survival (OS), progression-free survival (PFS), loco-regional relapse-free survival (LRRFS), and distant metastasis-free survival (DMFS) time than patients with low TAZ expression (*P* < 0.05). Multivariate analysis showed that high TAZ expression was an important prognostic factor for retinoblastoma patients. In addition, downregulation of TAZ expression significantly suppressed tumor cell proliferation by blocking the transition of the cell cycle from G_1_ to S phase.

**Conclusions:**

Our findings suggest that the high expression of TAZ plays a significant role in retinoblastoma’s aggressiveness, and predicts poor prognosis for patients with retinoblastoma.

## Background

Retinoblastoma is an aggressive malignancy of child retina with 1000 newly diagnosed cases annually in China [1]. Retinoblastoma affects the eyes of children at a very young age and accounts for 5 % of blindness in children [2]. Retinoblastoma often extends along the optic nerve into the brain and can easily distally metastasize. Although enucleation is necessary in some cases, recent advances have suggested chemoreduction as the primary modality of retinoblastoma management, to reduce the use of enucleation and improve the outcome of patients [3]. Therefore, exploring the underlying mechanism responsible for retinoblastoma development may contribute to finding new therapeutic targets for retinoblastoma treatment.

Transcriptional co-activator with PDZ-binding motif (TAZ) is a downstream factor of the Hippo signaling pathway, which controls cell proliferation and organ size in mammals [4, 5]. As soon as Hippo signaling is suppressed, TAZ phosphorylation decreases and it translocates into the nucleus. Then, TAZ binds to and interacts with several transcription factors including the TEA domain family members (TEAD) [6], T-box 5 (TBX5) [7] and paired box-8 (Pax8) [8], leading to activation of various target genes that are associated with cell proliferation and tissue growth. Recently, TAZ has attracted increased interest, because of its important roles in tumorigenesis. TAZ overexpression has been found in several human malignant tumors such as oral cancer [9], breast cancer [10], colon cancer [11] and hepatocellular carcinoma [12]. Moreover, TAZ overexpression promoted cell proliferation, migration and invasion, whereas downregulation of TAZ suppressed cell proliferation, migration and invasion in hepatocellular carcinoma cells [13] and neuroblastoma cells [14, 15]. However, the expression and the clinical significance of TAZ in retinoblastoma have not been elucidated, yet. In this study, we examined the expression of TAZ in retinoblastoma tissues, and analyzed its associations with clinicopathological features and prognosis of patients with retinoblastoma. We also investigated the role of TAZ in the proliferation of retinoblastoma cells by lentivirus-mediated knockdown of TAZ in a retinoblastoma cell line.

## Methods

### Human retinoblastoma cell lines

The human retinoblastoma cell lines, Y79 and WERI-Rb-1, were used in all experiments. WERI-Rb-1 cell line was purchased from the Institute of Biochemistry and Cell Biology of the Chinese Academy of Sciences (Shanghai, China). Y79 cell line was obtained from the American Type Culture Collection (ATCC, Rockville, MD, USA). All the cells were maintained in basic RPMI 1640 medium (Life Technologies, Grand Island, NY, USA) supplemented with 10 % fetal bovine serum (FBS; Life Technologies) and 1 % penicillin/streptomycin (Life Technologies) at 37 °C in a humidified atmosphere containing oxygen and 5 % CO_2_.

### Patients and specimens

We obtained human retinoblastoma samples from 50 patients and 5 normal retinas, and a complete set of follow-up data. All of the 43 retinoblastoma patients received enucleation or enucleation + chemotherapy +/− radiation therapy in the Department of Ophthalmology, Daping Hospital, Third Military Medical University, between February 2005 and November 2010. Of the 43 retinoblastoma patients there were 18 females and 25 males. The age of the patients was 0–7 years, with an average age of 2.6 years. All 43 retinoblastoma patients were confirmed histopathologically and staged based on the American Joint Commission for Cancer (AJCC) staging system [16]. The last follow-up date was at the end of December 2014. Other 7 retinoblastoma tissues obtained from patients received enucleation in the Department of Ophthalmology, Daping Hospital, Third Military Medical University, between November 2014 and March 2015. Five normal retinas obtained from patients who had died of conditions other than ophthalmologic diseases in Daping Hospital, Third Military Medical University. We have written informed consent from the donor or family members. In this study, all human participants and human specimens were approved by the ethics committee of the Third Military Medical University, and informed consent was obtained from all patients.

### Immunohistochemistry

For standard immunohistochemistry analysis, retinoblastoma tissue samples were fixed in 4 % formalin and dehydrated. Then, the tissues were embedded in paraffin. Immunohistochemistry assays were performed on 4-μmthick sections from each paraffin-embedded retinoblastoma specimen. After dewaxing and rehydrating the sections, antigen retrieval was performed in 10 mM citrate buffer at 100 °C for 5 min. Next, endogenous peroxidase activity was blocked using 3 % H_2_O_2_ in methanol for 10 min. Then, the sections were incubated at 4 °C overnight with mouse polyclonal anti-TAZ (1:50; BD biosciences, San Jose, CA, USA). After three washes with phosphate buffered saline (PBS), the sections were incubated with an anti-mouse secondary antibody for 1 h at room temperature, followed by detection using streptavidin-horseradish-peroxidase. Finally, the sections were counterstained with hematoxylin.

### Western blot analysis

Retinoblastoma cells were collected by centrifugation, and then 100 μg of protein extracts isolated with cold RIPA lysis buffer were separated by 10 % SDS-PAGE. After electrophoresis at 100 V for 2 h, proteins were transferred to polyvinylidenedifluoride (PVDF) membranes, which were then blocked with 5 % BSA in Tris-buffered saline containing 0.1 % Tween 20 (TBST) for 2 h at room temperature. Then, membranes were incubated with a primary antibody against human TAZ (1:500; BD biosciences), GAPDH (1:1000; Beyotime), CDK2 (1:500; Santa Cruz, Santa Cruz, CA, USA), CDK4 (1:500; Santa Cruz), CDK6 (1:500; Santa Cruz), Cyclin D1 (1:500; Santa Cruz), or Cyclin E (1:500; Abcam, Cambridge, MA, USA) at 4 °C overnight. After three 10-min washes with TBST, the membranes were incubated with the corresponding HRP-conjugated secondary antibody for 2 h at room temperature (1:1000; Santa Cruz). The targeted bands were visualized with ECL.

### RNA isolation and quantitative real-time PCR (qRT-PCR)

Total mRNA from the retinoblastoma and five normal retina tissues were extracted from snap-frozen tissue, which was extracted by Trizol reagent (Invitrogen, Carlsbad, CA, USA), and 2 μg of RNA were reverse transcribed with M-MLV Reverse Transcriptase (Promega, Madison, WI, USA) according to the manufacturer’s protocol. The mRNA expression level of *TAZ* was detected by the OneStep plus 7500 real-time PCR system (Bio-Rad, Hercules, CA, USA) using SYBER Green PCR Master mix (Takara Bio, Inc., Shiga, Japan). The *GAPDH* expression level was used as an endogenous control, and the mRNA level was calculated using the 2^−△△Ct^ method according to the manufacturer’s instructions.

### shRNA lentivirus vector construction and cell infection

The *TAZ*-specific short hairpin RNA (shTAZ) and *GFP*-specific short hairpin RNA (shGFP) were synthesized by GenePharma Co., Ltd. (Shanghai, China) and subcloned into the pLKO.1-puro lentivirus vectors. 293FT cells were cotransfected with a lentivirus vector with shTAZ or shGFP, and packaging plasmids using Lipofectamine 2000 (Life Technologies). After 48 h of transfection, viral supernatants were filtered and used to infect retinoblastoma cells. Stable cells expressing *TAZ* and control shRNA were generated after 5 days of culture with puromycin (4 μg/ml).

### Flow cytometry

Retinoblastoma cells (1 × 10^6^) were harvested and washed twice with cold PBS. Then, they were fixed with 70 % cold ethanol at 4 °C overnight. After two PBS washes, the cells were incubated with propidiumiodide (PI; BD Biosciences) and RNaseA for 30 min at room temperature. All samples were analyzed by FACSCanto II (BD Biosciences) with CellQuest software.

### MTT Assay

To analyze cell proliferation, retinoblastoma cells were seeded in 96-well plates at a density of 1000 cells per well. Absorbance values were measured by a microplate reader at 490 nm and the proliferation rates were analyzed by a cell growth curve. Each experiment was repeated independently at least three times.

### BrdU staining

Cells were grown on coverslips pre-coated with poly-L-lysine (PLL), and then incubated with 10 μg/ml BrdU (Sigma, St Louis, MO, USA) for 30 min. Next, the cells were washed with PBS, fixed with 4 % paraformaldehyde for 20 min, pre-treated with 1 mol/l HCl and blocked with 10 % goat serum for 1 h. Coverslips were then incubated with a monoclonal primary antibody against BrdU (1:200; Abcam) overnight at 4 °C, and then with Alexa FluorR® 594 goat anti-rat IgG secondary antibody for 2 h (Invitrogen). Hoechst (1:1000) was used for counterstaining. Images were captured from at least 10 microscope fields (Nikon 80i, Nikon Corporation, Tokyo, Japan) and analyzed using Image-Pro Plus software.

### Statistical analysis

All experiments were performed at least in triplicate and results were recorded. Statistical analysis was performed with the Statistical Package for the Social Sciences Version 16.0 software (SPSS, Chicago, IL, USA). The TAZ mRNA in retinoblastoma tissues and normal retina tissues was analyzed using t-test analysis. The clinicopathological features of retinoblastoma patients that correlated with TAZ low and high expression groups were determined by the Chi-square test. The TAZ expression in relation to overall survival (OS), progression-free survival (PFS), loco-regional relapse-free survival (LRRFS) and distant metastasis-free survival (DMFS) rates was analyzed using the Kaplan-Meier analysis and the log-rank test. The Cox regression analysis was used for univariate analysis and multivariate analysis of OS. *P* < 0.05 was considered statistically significant.

## Results

### Patient characteristics

Forty-three retinoblastoma patients were enrolled in this study and their characteristics are summarized in Table 1. At the time of diagnosis, the majority of the retinoblastoma patients were less than 5 years old and leukocoria was the most common sign of retinoblastoma (79.1 %). In the AJCC clinical classification (cTNM), there were 4.7 % in stage I, 27.9 % in stage II and 39.5 % in stage III. In addition, 34.9 % of the retinoblastomas had a large tumor base (>15 mm) and 44.2 % of the tumors were poorly differentiated. Moreover, the growth pattern of the retinoblastomas varied: 27.9, 46.5 and 11.6 % of the patients had endophytic growth, exophytic growth and mixed growth patterns, respectively.

**Table 1:** Patient demographics

Characteristic	No. of patients *n* = 43, n (%)
Patients	43 (100)
Age at presentation (y)	
< 5 y	39 (90.7)
≥ 5 y	4 (9.3)
Gender	
Male	18 (41.9)
Female	25 (58.1)
Hereditary pattern	
Sporadic	42 (97.7)
Familial	1 (2.3)
Laterality	
Unilateral	34 (79.1)
Bilateral	9 (20.9)
Unilateral (*n* = 34)	
Right	21 (61.8)
Left	13 (38.2)
First clinical presenting signs	
Leucocoria	34 (79.1)
Strabismus	5 (11.6)
Proptosis	3 (7.0)
Swelling	1 (2.3)
cTNM clinical classification	
T classification	
T1	10 (23.3)
T2	14 (32.6)
T3	11 (25.6)
T4	8 (18.6)
N classification	
N0	20 (46.5)
N1	17 (39.5)
N2	6 (14.0)
M classification	
M0	31 (72.1)
M1	12 (27.9)
cTNM stage	
I	2 (4.7)
II	12 (27.9)
III	17 (39.5)
IV	12 (27.9)
Largest tumor base (mm), mean (median, range)	11.3 (5–26)
≤ 15 mm	28 (58.1)
> 15 mm	15 (41.9)
Tumor thickness (mm), mean (median, range)	8.6 (9, 3–16)
≤ 10 mm	25 (58.1)
> 10 mm	18 (41.9)
Growth pattern	
Endophytic	12 (27.9)
Exophytic	20 (46.5)
Mixed	5 (11.6)
Diffuse infiltrating	6 (14.0)
Differentiation	
Well	9 (20.9)
Moderate	15 (34.9)
Poorly	19 (44.2)
Treatment	
Eye enucleated	19 (44.2)
Enucleated + chemotherapy	22 (51.2)
Enucleated + chemotherapy + radiation therapy	2 (4.7)
Outcomes	
Death	14 (32.6)
Survival	29 (67.4)

### TAZ expression in retinoblastoma and normal retina tissues

**Fig. 1:**
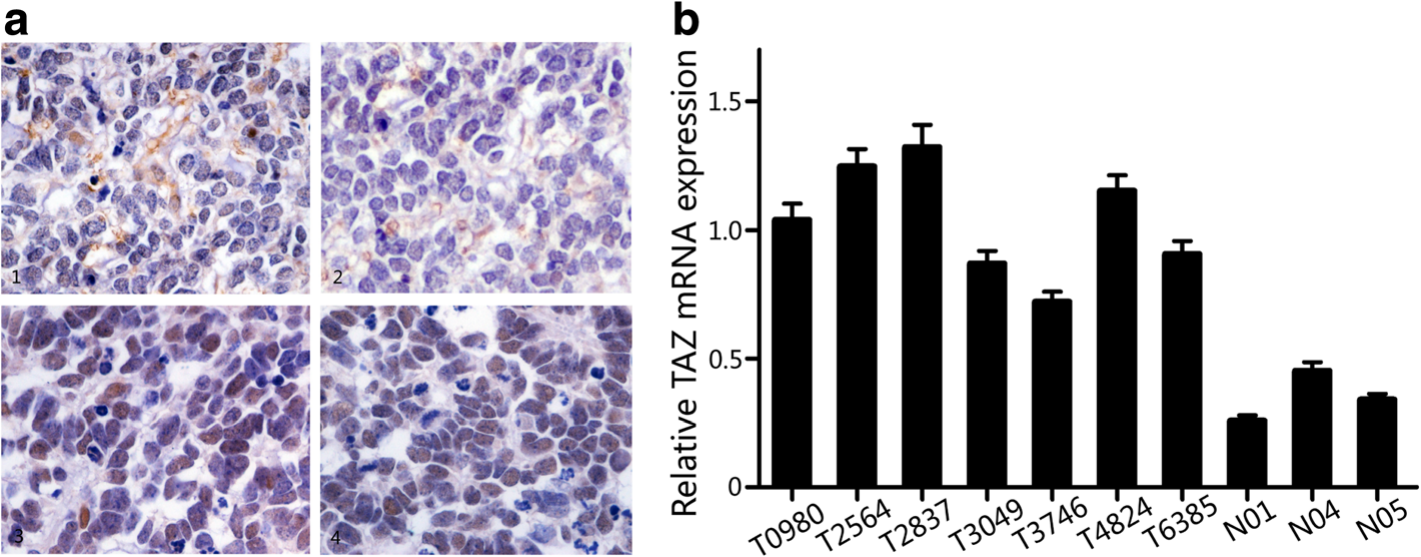
TAZ is commonly expressed in retinoblastoma **a** 1 and 2) Low immunohistochemical staining of TAZ in retinoblastoma. **a** 3 and 4) High immunohistochemical staining of TAZ in retinoblastoma. **b**) mRNA level of TAZ in seven human retinoblastoma tissues and three normal retina tissues were analyzed by quantitative real time-PCR

TAZ expression was investigated in retinoblastoma specimens by immunohistochemistry (Fig. 1a). TAZ was highly expressed in 65.1 % (28/43) of the retinoblastoma samples. Next, RT-PCR was used to examine the *TAZ* mRNA level in seven human retinoblastoma tissues (T0980, T2564, T2837, T3049, T3746, T4824, T6385) and three human normal retina tissues (N01, N04, N05). The five normal retina tissues all showed TAZ mRNA expression. As shown in Fig. 1b, *TAZ* mRNA had higher expression in retinoblastoma tissues than in normal retina tissues (*P* = 0.038). GAPDH mRNA expression of every tissue was used as control for itself.

### Downregulation of TAZ inhibits cell proliferation in retinoblastoma cells

**Fig. 2:**
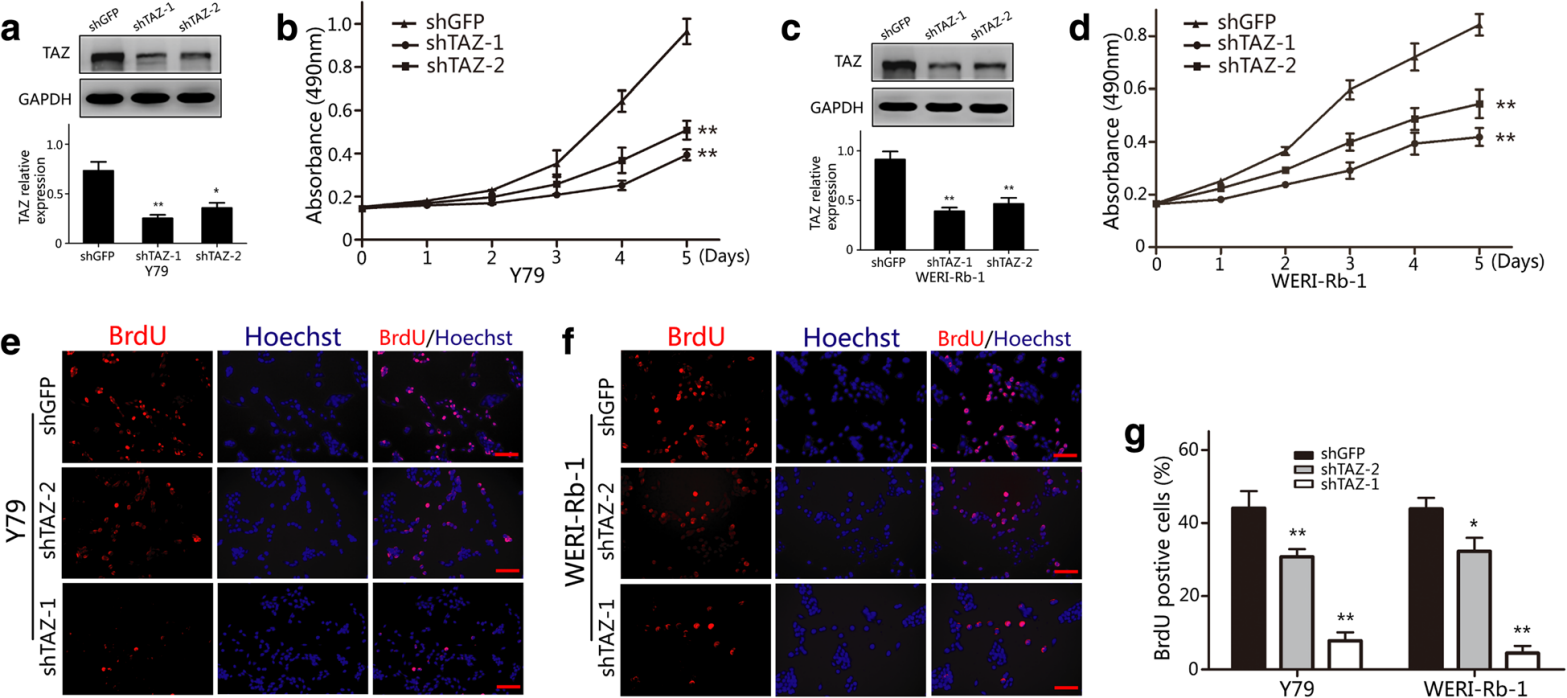
Downregulation of TAZ inhibits retinoblastoma proliferation **a**) Western blot assay was used to analyze TAZ expression in TAZ-knockdown Y79 cells. **b**) The effect of TAZ on the proliferation of Y79 cells was determined by the MTT assay. **c**) Western blot assay was used to analyze TAZ expression in TAZknockdown WERI-Rb-1 cells. **d**) The effect of TAZ on the proliferation of WERI-Rb- 1 cells was determined by the MTT assay. **e**–**g**) Images and quantification of Y79 and WERI-Rb-1 cells positive for BrdU staining. GAPDH was used as a loading control; student’s t-test was carried out. All data are shown as mean ± SD, *P< 0.05, **P< 0.01

The effect of TAZ on retinoblastoma cell lines was determined through lentivirus-mediated knockdown of TAZ. Western blot analysis showed that the level of TAZ expression significantly decreased in WERI-Rb-1 and Y79 retinoblastoma cells (Fig. 2a, c). Next, we analyzed the role of TAZ in the proliferation of retinoblastoma cells by the MTT assay. The results showed that the proliferation of retinoblastoma cells was substantially suppressed after TAZ knockdown (Fig. 2b, d). Furthermore, retinoblastoma cells with TAZ knockdown showed a 39 % reductionin BrdU-positive cells compared with the shGFP control group (Fig. 2e–g). These results suggest that TAZ promotes proliferation in retinoblastoma cell lines.

### Downregulation of TAZ blocks cell cycle progression in retinoblastoma cells

**Fig. 3:**
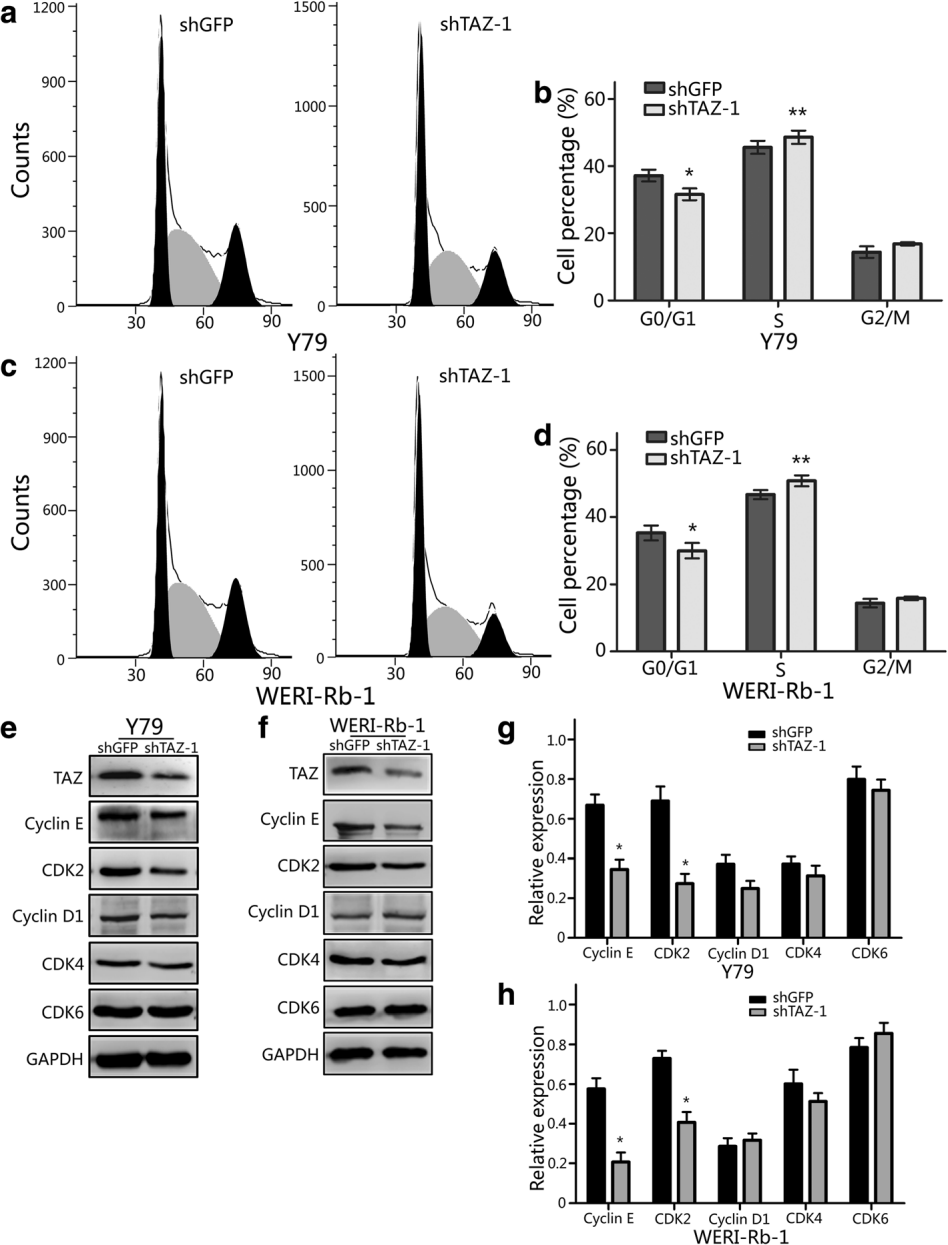
Downregulation of TAZ blocks the cell cycle transition from G1 to S phase **a**) The cell cycle of TAZ-knockdown Y79 cells was analyzed by flow cytometry. **b**) The effects of TAZ on the cell cycle of Y79 cells. **c**) The cell cycle of TAZ-knockdown WERI-Rb-1cellswas analyzed flow cytometry. **d**) The effects of TAZ on the cell cycle of WERI-Rb-1 cells. **e** and **f**) Western blot analysis of Cyclins and CDKs expression in TAZ-knockdown Y79 and WERI-Rb-1 cells. **g** and **h**) Quantitative analysis of Cyclins and CDKs expression in TAZ-knockdown Y79 and WERI-Rb-1 cells; GAPDH was used as a loading control; student’s t-test was carried out. All data are shown as mean ± SD, *P< 0.05, **P< 0.01

To further assess the effects of TAZ expression on retinoblastoma, we used flow cytometry to analyze the cell cycle of retinoblastoma cells (Fig. 3a–d). The results showed that the number of cells in G_1_ phase in both WERI-Rb-1 and Y79 cells increased after TAZ knockdown. Moreover, we examined the expression levels of Cyclins and Cyclin-dependent kinases (CDKs), which are crucial for regulating the transition from G_1_ to S phase, by western blot analysis. We found that the expression of Cyclin E and CDK2 was downregulated in retinoblastoma cells with TAZ knockdown, but no obvious changes were observed in Cyclin D1, CDK4 and CDK6 expression (Fig. 3e–h). These findings suggest that TAZ promotes cell cycle progression via upregulation of Cyclin E and CDK2.

### Correlation between TAZ expression and clinicopathological features

The correlation between clinicopathological features and the expression level of TAZ is showed in Table 2. The results showed that TAZ high expression significantly correlated with regional lymph node classification (*P* = 0.013), largest tumor base (*P* = 0.045), and differentiation (*P* = 0.019), but no significant correlation was observed between TAZ expression levels and the patients’ age, gender, tumor enucleated location or tumor thickness.

**Table 2:** Correlation between clinicopathological features and expression of TAZ in retinoblastoma patients

Feature	All patients *n* = 43, *n* (%)	Expression of TAZ		*P* value
		High	Low	
Age at presentation (y)		28	15	0.602
< 5 y	39 (90.7)	26	13	
≥ 5 y	4 (9.3)	2	2	
Gender		28	15	0.338
Male	18 (41.9)	10	8	
Female	25 (58.1)	18	7	
Tumor enucleated location		28	15	1.000
Right	25 (58.1)	16	9	
Left	18 (41.9)	12	6	
cTNM classification				
T classification		28	15	0.116
T1 + 2	24	13	11	
T3 + 4	19	15	4	
N classification		28	15	0.013*
N0	20	9	11	
N1 + 2	23	19	4	
cTNM stage		28	15	0.184
I + II	14	7	7	
III + IV	29	21	8	
Largest tumor base (mm)		28	15	0.045*
≤ 15 mm	28 (65.1)	15	13	
> 15 mm	15 (34.9)	13	2	
Tumor thickness (mm)		28	15	0.750
≤ 10 mm	25 (58.1)	17	8	
> 10 mm	18 (41.9)	11	7	
Differentiation		28	15	0.019*
Well and moderate	24 (55.8)	12	12	
Poorly	19 (44.2)	16	3	

*P < 0.05

### Association of TAZ expression with OS, PFS, LRRFS and DMFS

**Fig. 4:**
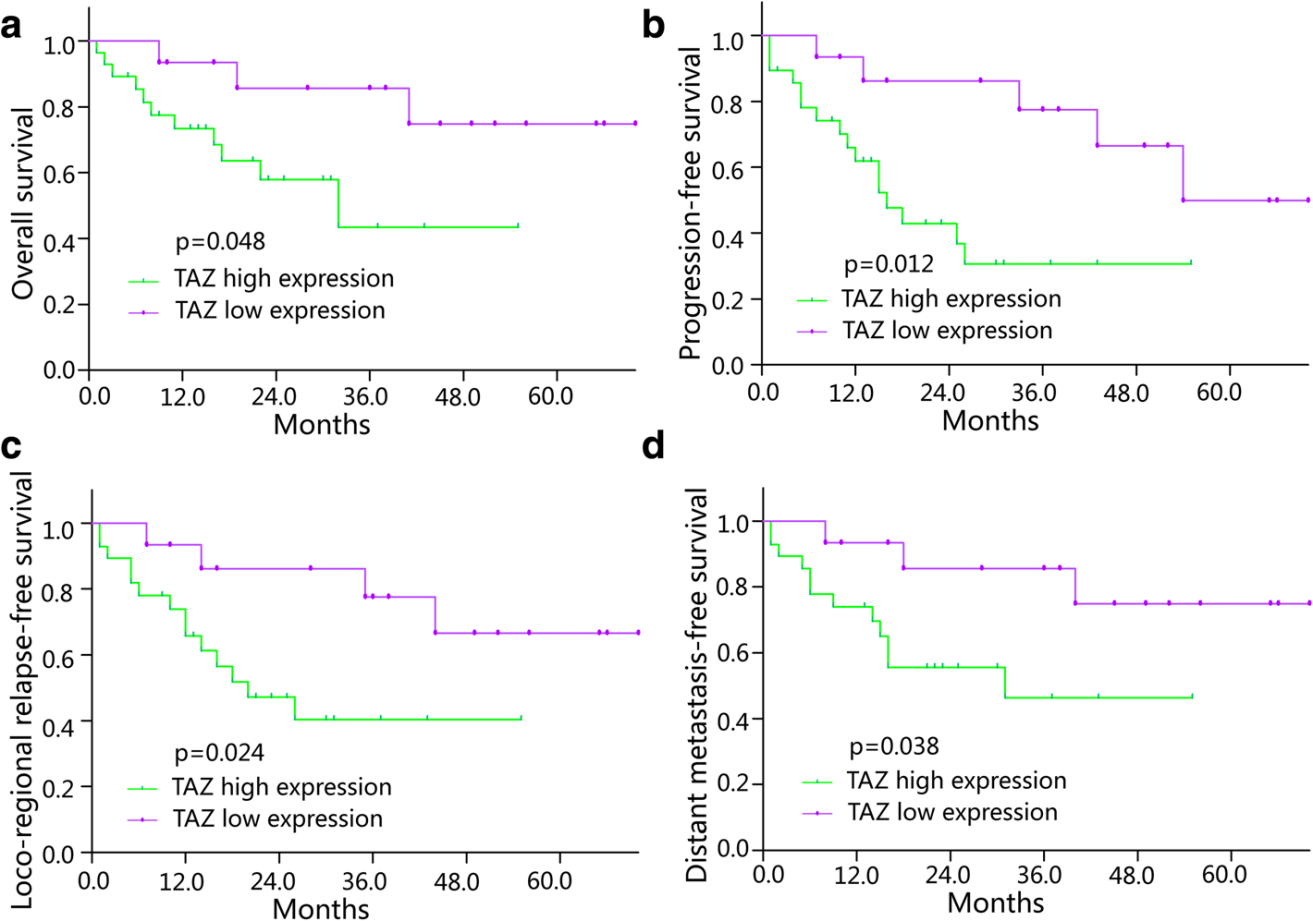
Correlation between TAZ expression and OS, PFS, LRRFS and DMFS in retinoblastoma patients **a**) The Kaplan-Meier analysis of OS for retinoblastoma patients with the log-rank test. **b**) The Kaplan-Meier analysis of PFS for retinoblastoma patients with the logrank test. **c**) The Kaplan-Meier analysis of LRRFS for retinoblastoma patients with the log-rank test. **d**) The Kaplan-Meier analysis of DMFS for retinoblastoma patients with the log-rank test. *P< 0.05

Until Dec 2014, there were 21 patients with progression, 18 patients with loco-regional relapse and 15 patients with distant metastasis, respectively. Kaplan-Meier analysis showed that retinoblastoma patients with TAZ high expression had shorter OS (*P* = 0.048), PFS (*P* = 0.012), LRRFS (*P* = 0.012) and DMFS (*P* = 0.038) than those with TAZ low expression (Fig. 4). The relationship between the 5-year OS, PFS, LRRFS and DMFS of retinoblastoma patients and the levels of TAZ expression are shown in Table 3. Moreover, univariate analysis demonstrated that primary tumor classification (*P* = 0.002), regional lymph node classification (*P* = 0.012), clinical stage (*P* = 0.026), largest tumor base (*P* = 0.040) and differentiation (*P* = 0.002) were associated with a poor prognosis (Table 4). Furthermore, the multivariate Cox regression analysis results revealed that advanced clinical stage and TAZ high expression were associated with poor OS, PFS, LRRFS and DMFS (Table 5).

**Table 3:** Association of TAZ expression with 5-year survival in retinoblastoma patients

Group	5-year OS		5-year PFS		5-year LRRFS		5-year DMFS	
	Rate (%)	*P* value	Rate (%)	*P* value	Rate (%)	*P* value	Rate (%)	*P* value
TAZ								
High expression	60.7	0.048*	42.9	0.012*	50.0	0.024*	57.1	0.038*
Low expression	80.0		66.7		73.3		80.0	

*P < 0.05

**Table 4:** Cox univariate analysis of overall survival

Variables	SE value	Hazard ratio	Risk ratio (95 % CI)	*P* value
Age (≥5 y)	1.043	0.835	0.108–6.451	0.863
Gender (female)	0.558	0.806	0.270–2.409	0.700
Tumor enucleated location (left)	0.536	0.702	0.246–2.006	0.702
Primary tumor (cT3 + T4)	0.680	0.122	0.032–0.462	0.002*
Regional lymph node (cN1 + N2)	0.793	0.135	0.028–0.638	0.012*
cTNM stage (III + IV)	1.043	0.098	0.013–0.760	0.026*
Largest tumor base (>15 mm)	0.591	0.297	0.093–0.948	0.040*
Tumor thickness (>10 mm)	0.545	0.668	0.230–1.943	0.459
Differentiation (poorly)	0.789	0.085	0.018–0.401	0.002*
TAZ high expression	0.601	0.314	0.112–1.129	0.042*

*P < 0.05

**Table 5:** Cox multivariate analysis for retinoblastoma patients

Variables	SE value	Hazard ratio	Risk ratio (95 % CI)	*P* Value
OS				
cTNM stage (III + IV)	1.065	0.085	0.011–0.687	0.021*
TAZ high expression	0.756	0.226	0.051–0.994	0.049*
PFS				
cTNM stage (III + IV)	1.097	0.030	0.003–0.254	0.001*
TAZ high expression	0.692	0.144	0.037–0.559	0.005*
LRRFS				
cTNM stage (III + IV)	1.062	0.056	0.007–0.452	0.007*
TAZ high expression	0.710	0.195	0.049–0.783	0.021*
DMFS				
cTNM stage (III + IV)	1.058	0.084	0.011–0.672	0.019*
+TAZ high expression	0.735	0.228	0.054–0.960	0.044*

*P < 0.05

## Discussion

Accumulated research has revealed that TAZ plays a critical role in cell proliferation, survival and tissue growth control [9, 17, 18]. Recently, high expression of TAZ has been reported to be closely associated with carcinogenesis in several human malignant cancers, such as oral squamous cell carcinoma [19], breast cancer [20], lung cancer [21], hepatocellular carcinoma [12] and ovarian cancer [22]. However, whether TAZ is highly expressed in retinoblastoma remains unclear. To obtain more insight into the expression status and clinical significance of TAZ in retinoblastoma, we performed immunohistochemistry staining to explore the expression level of TAZ and to analyze its association with clinicopathological parameters of retinoblastoma patients. Our results demonstrated that TAZ was highly expressed in the majority of retinoblastoma specimens and its expression correlated with regional lymph node classification largest tumor base, and tumor differentiation. More importantly, we also found that the TAZ expression was an important biomarker for prognosis of patients with retinoblastoma. By knocking down TAZ expression with lentivirus-mediated shRNA interference, we showed that downregulation of TAZ effectively decreased retinoblastoma cell proliferation. Thus, our data suggest that high TAZ levels promote retinoblastoma progression.

Retinoblastoma is the most common eye cancer in children, which is caused by mutation of the *RB1* gene [23–25]. It occurs in approximately 1 per 15,000–20,000 live births and there are about 9000 new cases every year [26]. Retinoblastoma was the first tumor to emphasize the genetic aberrations in carcinogenesis. Despite good understanding of its etiology, about 40–70 % of children suffering from retinoblastoma die in developing countries in Africa and Asia [24]. Therefore, it is necessary to further dissect the underlying mechanisms of retinoblastoma initiation and development, to find valuable therapeutic targets.

In this study, we found that TAZ expression was associated with regional lymph node classification, largest tumor base, and tumor differentiation, suggesting that TAZ is involved in retinoblastoma development. More importantly, we demonstrated that high TAZ expression was significantly associated with shorter OS, PFS, LRRFS and DMFS time in patients with retinoblastoma. The results form Kaplan-Meier analysis and the log-rank test showed that patients whose tumors had higher TAZ expression tended to have a significantly worse overall survival rate, indicating that a high TAZ level is a biomarker of poor prognosis for patients with retinoblastoma. In addition, the Cox proportional hazards regression model showed that high TAZ expression was a biomarker of worse OS. Furthermore, our data showed that knockdown of TAZ expression in retinoblastoma cells blocked the cell cycle transition from G_1_ to S phase and decreased the protein expression of Cyclin E and CDK2, again suggesting that TAZ participates in the regulation of cell cycle pathways in retinoblastoma cells. In summary, we demonstrated that TAZ promotes retinoblastoma growth via downregulating Cyclin E and CDK2 expression. Therefore, our data showed that TAZ expression represents a potential biomarker for predicting the prognosis of patients with retinoblastoma and for identifying patients in higher risk of death.

## Conclusions

In summary, our results proved that high expression of TAZ promoted proliferation of retinoblastoma cells and was associated with poor prognosis for patients with retinoblastoma, suggesting that TAZ may serve as a valuable biomarker for predicting the clinical prognosis of patients with retinoblastoma and is a potential therapeutic target.

## Abbreviations

TAZ: Transcriptional co-activator with PDZ-binding motif
ATCC: American Type Culture Collection
FBS: Fetal bovine serum
AJCC: American Joint Commission for Cancer
PBS: Phosphate buffered saline
SDS-PAGE: Sodium dodecyl sulfate-polyacrylamide gel electrophoresis
TBST: Tris-buffered saline containing 0.1 % Tween 20
PVDF: Polyvinylidenedifluoride
shTAZ: TAZ-specific short hairpin RNA
shGFP: GFP-specific short hairpin RNA
PI: Propidiumiodide
MTT: 3-(4,5-dimethylthiazol-2-yl)-2,5-diphenyltetrazoliumbromide
DMSO: Dimethylsulfoxide
PLL: Poly-L-lysine
SPSS: Social sciences version 16.0 software
OS: Overall survival
PFS: Progression-free survival
LRRFS: Loco-regional relapse-free survival
DMFS: Distant metastasis-free survival
cTNM: Tumor-node-metastasis staging by clinical examination
CDKs: Cyclin-dependent kinases
